# The Art Amateur for October

**Published:** 1894-11

**Authors:** 


THE ART AMATEUR for October is out. Previous issues have been noticed in this journal from time to time, and its advertisement regularly appears.

In the October number: "Meditation," by Charles Sprague Pearce, a charming picture of a girl whose thoughts have drifted from the book which she has been reading, and "Peonies," by Paul de Longpré, one of the most popular of the flower-painters of the day, appear, admirably reproduced, as color studies in *The Art Amateur*. If the first of these has a slight tinge of pleasant sadness, the frontispiece more than counterbalances it—a child, almost smiling, holding up a bunch of "Cherries" (an engraving by Baude after the pastel by John Russell). Other specially notable illustrations in the number are a water-color study of a bull by Rosa Bonheur, "Juliet" (delicately engraved from the painting by Wagrez), "Normandy Fisherwomen" (a crayon by Feyen Perrin), Sir Edwin Landseer's "King Charles Spaniels," portraits of the late George Inness and Wm. M. Chase, etc., etc. But it is in the practical articles and designs that the reader of this magazine is always most interested, and this month he (or she) is particularly fortunate in articles on "Landscape Painting in Water-Colors," "Flower Drawings in Pen-and-ink," "Use of the Stamp in Pastel," "Drawing from Life," "Flowers and Plants in Decoration," "Talks on Embroidery," "Common Sense in China Painting," "What Colors Will Mix" (for china painters), and a host of other timely papers for the painter, draughtsman, wood-carver, needle-worker, and other art producers. Among the designs the fish and game-plates by Charles Volkmar are remarkably beautiful specimens of how a dinner service can be decorated, and the wood-worker will be especially delighted with the detailed scheme for a panel of a book-case (the complete design appearing elsewhere in the magazine). The other supplements have numerous designs for embroidery, china painting, glass decoration, etc., etc. Of more general interest, perhaps, are an extended description of Mr. Inness's work, a continuation of Theodore Child's articles on The National Gallery (London), some further "Secrets of the Art Trades," an account of the Summer School at Shinnecock Hill (with some of Mr. Chase's valuable hints to his pupils, which are to be continued from month to month), a notice of the St. Louis Exposition, etc. The Ex-Libris department is well represented and there is another of the papers on Christian Iconography and Symbolism, copiously illustrated. We have touched upon only a few of many features of *The Art Amateur*, whose practical usefulness becomes more evident with every succeeding issue. It goes without saying that the editor's comments in his "Note-Book" on current art-topics are what every regular reader will turn to first. (Price, 35 cents.) Montague Marks, Publisher, 23 Union Square, New York.

